# A Nonparametric Model for Multi-Manifold Clustering with Mixture of Gaussians and Graph Consistency

**DOI:** 10.3390/e20110830

**Published:** 2018-10-29

**Authors:** Xulun Ye, Jieyu Zhao, Yu Chen

**Affiliations:** Institute of Computer Science and Technology, Ningbo University, Ningbo 315211, China

**Keywords:** multi-manifold clustering, Dirichlet process mixture model, mixture of Gaussians, graph theory

## Abstract

Multi-manifold clustering is among the most fundamental tasks in signal processing and machine learning. Although the existing multi-manifold clustering methods are quite powerful, learning the cluster number automatically from data is still a challenge. In this paper, a novel unsupervised generative clustering approach within the Bayesian nonparametric framework has been proposed. Specifically, our manifold method automatically selects the cluster number with a Dirichlet Process (DP) prior. Then, a DP-based mixture model with constrained Mixture of Gaussians (MoG) is constructed to handle the manifold data. Finally, we integrate our model with the *k*-nearest neighbor graph to capture the manifold geometric information. An efficient optimization algorithm has also been derived to do the model inference and optimization. Experimental results on synthetic datasets and real-world benchmark datasets exhibit the effectiveness of this new DP-based manifold method.

## 1. Introduction

Over the past decades, clustering has been the most fundamental task in many computer vision and data mining applications [1,2], e.g., image/motion segmentation [3,4], community detection [5], feature selection [6] and biological/network information analysis [7,8]. However, most of the conventional clustering methods assume that data samples are scattered in the feature space, which ignores the intrinsic underlying data structure that many real datasets have [3,9]. To overcome this problem, various manifold-based clustering (multi-manifold clustering) methods have been proposed and developed. Compared to the conventional clustering method, which regards the cluster as the data points with small distances between cluster members or dense areas of the feature space, the multi-manifold approach aims to gather the given data points into disparate groups, which come from different underlying submanifolds [10].

Unlike the conventional clustering methods [11,12], multi-manifold clustering can be classified into two different categories, the linear method and the nonlinear method [13]. In the first category, linear methods (also known as subspace clustering) construct the multi-manifold clustering by assuming that the underlying cluster can be well approximated by a union of low dimensional linear manifolds [14]. For example, Gholami [14] and Vidal [15] used a linear function to fit the underlying submanifold and cluster the clusters with the mixture model. Sparse Subspace Clustering (SSC)- [16], Low-Rank Representation (LRR)- [17] and Least Squares Regression (LSR)-based [18] methods approach the linear manifold clustering problem by finding a sparse representation of each point in terms of other data points. After forming a similarity graph with the learned sparse representation, spectral clustering methods are used to cluster data into distinctive clusters. As an expanding framework of the linear multi-manifold clustering methods, non-linear algorithms can be naturally applied to linear and/or nonlinear manifolds. For example, the *K*-manifold clusters the nonlinear subspace dataset by expanding the conventional *K*-means with geodesic distance [19]. Spectral Multi-Manifold Clustering (SMMC) integrates the local geometric information within the subspace clustering framework to handle the manifold structure [13], Multi-Manifold Matrix Decomposition for Co-clustering (M3DC) handles the manifold dataset by considering the geometric structures of both the sample manifold and the feature manifold simultaneously [20]. Recently, the state-of-the-art method may be deep subspace clustering, which assembles the deep framework and the conventional subspace clustering method [21,22].

However, a drawback of most conventional manifold clustering methods is that the clustering accuracy depends on the cluster number, which is always unavailable in advance [23]. To overcome this model selection problem, one category of the most widely-studied methods is that equipping the conventional methods with a Dirichlet process prior, e.g., Dirichlet Process Mixture (DPM) models [24,25], Multilevel Clustering with Context (${\mathrm{MC}}^{2}$) [26] and Dirichlet Process Variable Clustering (DPVC) [27]. Since the distributions adopted in these nonparametric models are defined in the Euclidean space, those conventional Dirichlet process clustering methods suffer difficulty when dealing with the manifold data. To overcome this problem, many manifold DP clustering models have been proposed. Wang [28] and Gholami [14] assumed that the submanifold is lying on the linear manifold and can be fitted with the hyperplane. Straub et al. [29,30] defined the Gaussian distribution on the sphere surface and introduced an auxiliary indicator vector zwith a DP prior. More than the sphere manifold, Simo et al. [31] expanded the distribution to the manifold space with the logarithmic and exponential mapping. Although these models are quite powerful and have been widely studied in many applications, they have their drawbacks when the manifold structure is not prespecified [31]. For example, the DP-space and temporal subspace clustering model is an expanding method of the linear manifold clustering method. It lacks the capability to handle a non-linear manifold dataset. In the geodesic mixture model, the logarithmic and exponential mapping algorithms [32,33] used in this model depend mainly on the pre-defined geometric structure, which is always unavailable. For the sphere mixture model, the sphere manifold has not been extended to arbitrary manifolds [31].

**Figure 1:**
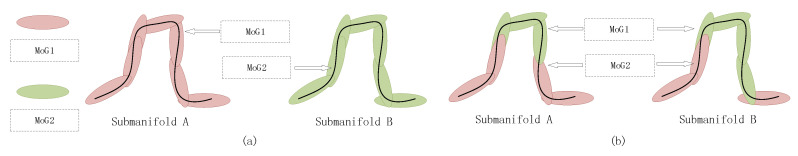
Illustration of manifold modeling. Mixture of Gaussians (MoG) distributions are demonstrated to model the submanifolds. (**a**) demonstrates two ideal results using two MoG distributions to model the submanifold; (**b**) demonstrates a result where there is no geometric information and mean constraint, in which Gaussian distributions in the two MoGs may be scattered into two submanifolds.

In this paper, we investigate the manifold clustering method with no prespecified manifold structure and cluster number in the DPM framework. In order to model the complicated manifold cluster distributions, we integrate the original DPM with the conventional Mixture of Gaussians (MoG) [34,35] to handle the manifold distribution (Figure 1a). Furthermore, we also notice that an unconstrained MoG distribution fails to capture the manifold geometrical information (Figure 1b). Inspired by the previous study [23,36], we regularize our model with a *k*-nearest neighbor graph. To form a meaningful cluster, in which samples from the same cluster are closed and related, we constrain the MoG mean with a Mahalanobis distance.

The main contributions are as follows:

- A constrained MoG distribution has been applied to model the non-Gaussian manifold distribution.
- We integrate the graph theory with DPM to capture the manifold geometrical information.
- The variational inference-based optimization framework is proposed to carry out the model inference and learning.

The organization of our paper proceeds as follows. In Section 2, we review the background knowledge of the Dirichlet process mixture model. Simultaneously, we present the generation procedure of the proposed manifold Dirichlet process mixture model and give the variational expectation maximization inference algorithm. Experimental comparisons will be presented in Section 3. In Section 4, we give the detailed discussions and present the limitations and advantages. Section 5 concludes the paper.

## 2. Materials and Methods

In this section, we firstly review the basic concept of the Dirichlet Process Mixture (DPM) model. Then, we propose the multi-manifold clustering method by equipping DPM with MoG and the *k*-nearest neighbor graph. In our method, the Dirichlet process is used to generate the suitable cluster number. MoG and the *k*-nearest neighbor graph are applied to model the non-Gaussian manifold distribution and capture the manifold geometric information. Finally, variational inference is derived to do the model inference and learning.

### 2.1. Dirichlet Process Mixture Model

The Dirichlet Process Mixture (DPM) model is an approach that extends the mixture model by introducing a Dirichlet process prior within the Bayesian framework. In DPM, we firstly sample a prior distribution *G* from the Dirichlet process and then sample the likelihood parameters ${\left\{\theta_{n}\right\}}_{n=1}^{N}$ from *G*. With the sampled likelihood parameters, observation data $x_{n}$ can be generated from the likelihood distribution $F(x|\theta_{n})$. This procedure can be concluded as follows:

(1) $$\begin{array}{c} G|G_{0}\left(\lambda \right)∼DP\left(G_{0}\left(\lambda \right),\alpha \right)\\ \theta_{n}|G∼G\;n=1,2,3,\ldots ,N\\ x_{n}∼F\left(x|\theta_{n}\right)\;n=1,2,3,\ldots ,N, \end{array}$$

where $F(x|\theta_{n})$ is a likelihood distribution and $G_{0}$ is a base distribution. $x_{n}$ is the observation sample.

By integrating out *G*, the joint distribution of the likelihood parameters ${\left\{\theta_{n}\right\}}_{n=1}^{N}$ exhibits a clustering effect. Suppose that we have N−1 parameters ${\left\{\theta_{n}\right\}}_{n=1}^{N-1}$ sampled from our Dirichlet process. We then have the following probability for the *N*-th value of θ.

(2) $$\begin{array}{c} p\left(\theta_{N}|{\left\{\theta_{n}\right\}}_{n=1}^{N-1}\right)=\frac{\alpha G_{0}}{\alpha +N-1}+\sum_{i=1}^{I}\frac{n_{i}\delta \left(i\right)}{\alpha +N-1}, \end{array}$$

where $n_{i}$ denotes the θ frequency of occurrence in ${\left\{\theta_{n}\right\}}_{n=1}^{N-1}$ and δ(j) represents the delta function. *I* denotes the number of unique values in ${\left\{\theta_{n}\right\}}_{n=1}^{N}$. (2) reveals the fact that a new sample $\theta_{n}$ is either generated from a new cluster with probability $G_{0}$ or extracted from the existing clusters ${\left\{\theta_{n}\right\}}_{n=1}^{N-1}$ with probability $n_{i}/\left(\alpha +N-1\right)$.

### 2.2. Our Proposed Method

In this section, we expand the original DPM model to a multi-manifold clustering framework named the Similarity Dirichlet Process Mixture (SimDPM) model. The main notations and descriptions used in our method are summarized in Table 1.

As we have debated, DPM is unable to model the manifold dataset since the conventional likelihood distribution $F(x|\theta_{n})$ is defined in the Euclidean space or prespecified manifold. To overcome this problem, we approximate the manifold distribution with MoG (Figure 1a). Then, we construct the sample generation process with two phases, a single Gaussian distribution and a mixture of Gaussians distribution. The reason we generate the data with both the single Gaussian distribution and the MoG distribution is that some simple submanifolds and non-manifold clusters can be modeled by the single Gaussian distribution.

**Figure 2:**
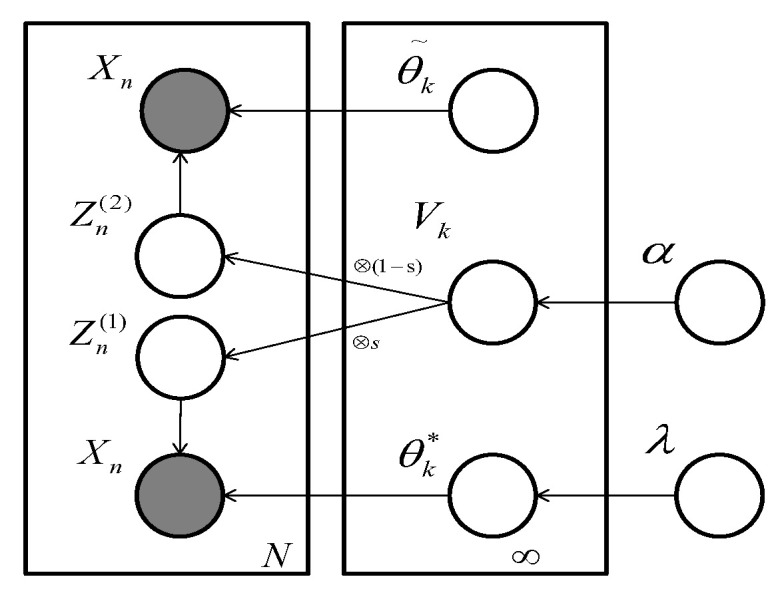
Probability Graph Model (PGM) representation of the Similarity Dirichlet Process Mixture (SimDPM) model. Nodes denote the random variables. In our framework, observations are generated from two phases, a fully-Bayesian procedure and a constrained MoG model.

Suppose that we are given *N* observation samples $X={\left\{x_{n}\right\}}_{n=1}^{N}$ where $x_{n}\in R^{D}$. Given the additional parameters of the MoG distribution, we assume the following generative process for each observation data $x_{i}$:

1. For i=1,2,3,…; draw $v_{i}|\alpha ∼Beta\left(1,\alpha \right)$
2. For i=1,2,3,…; draw $\theta_{i}^{*}|G_{0}∼G_{0}$
3. For every data point *i*:
  (a) Choose $z_{i}^{\left(1\right)}|v∼mult\left(s\pi \left(v\right)\right)$
  (b) Choose $z_{i}^{\left(2\right)}|v∼mult\left(\left(1-s\right)\pi \left(v\right)\right)$
  (c) Draw $x_{i}|z_{i}^{\left(1\right)}∼N\left(x|\theta_{z_{i}^{\left(1\right)}}^{*}\right)$
  (d) Draw $x_{i}|z_{i}^{\left(2\right)}∼MoG\left(x|{\tilde{\theta }}_{z_{i}^{\left(2\right)}}\right)$

where Beta(1,α) is a beta distribution with parameter one and α, mult(π(v)) is a categorical distribution parameterized by π(v), v and π(v) are vectors with $v={\left\{v_{k}\right\}}_{k=1}^{\infty }$ and $\pi \left(v_{i}\right)=v_{i}\prod_{j=1}^{i-1}\left(1-v_{j}\right)$ and $G_{0}$ is a normal-Wishart distribution with parameter $\lambda =(u_{0},c_{0},W_{0},v_{0})$ where $u_{0}\in R^{D}$ $W_{0}\in R^{D\times D}$, $\theta_{z_{i}^{\left(1\right)}}^{*}$ is a Gaussian distribution parameterized by $u_{z_{i}^{\left(1\right)}}\in R^{D},\delta_{z_{i}^{\left(1\right)}}\in R^{D\times D}$. ${\tilde{\theta }}_{z_{i}^{\left(2\right)}}$ is the MoG distribution with parameter ${\tilde{u}}_{z_{i}^{\left(2\right)}}\in R^{D},{\tilde{\delta }}_{z_{i}^{\left(2\right)}}\in R^{D\times D},{\tilde{\pi }}_{z_{i}^{\left(2\right)}}\in R^{M}$ where *M* denotes the mixture number in every ${\tilde{\theta }}_{z_{i}^{\left(2\right)}}$. Tradeoff parameter *s* denotes how likely the observation sample $x_{n}$ is sampled from a single Gaussian distribution. The corresponding probability graph model representation of manifold DPM can be described as Figure 2.

To form a meaningful cluster (samples from the same cluster are closely related) and respect the manifold geometrical information, we constrain the MoG mean with:

$$\frac{1}{2}{\left({\tilde{u}}_{k,m}-{\tilde{u}}_{k,m-1}\right)}^{T}{\tilde{\delta }}_{k,m}^{-1}\left({\tilde{u}}_{k,m}-{\tilde{u}}_{k,m-1}\right)\epsilon ,\;m1,$$

and use a *k*-nearest neighbor graph to regularize the posterior probability inspired by [23], in which the graph Laplacian is used to capture the geometric information that has been missed by the MoG distribution.

(3) $$\begin{array}{c} R=\sum_{k=1}^{\infty }p{\left(k|X\right)}^{T}Lp\left(k|X\right), \end{array}$$

where $p\left(k|X\right)={\left\{p\left(k|x_{n}\right)\right\}}_{n=1}^{N}$ is the posterior probability. *L* is the graph Laplacian constructed by the *k*-nearest neighbor graph [13]. Note that the constraint of ${\tilde{u}}_{k,m}$ depends only on the previous ${\tilde{u}}_{k,m-1}$, but not on ${\tilde{u}}_{k,m+1}$. Below, we characterize the *k*-nearest neighbor graph $L^{k}$. Given the unlabeled data *X*, for any point $x_{i}$, we sort the rest of the data samples and select the top-*k* nearest neighbors. If node $x_{j}$ is in the top-*k* nearest points of node $x_{i}$, we set:

$$L_{i,j}^{k}=e^{\frac{-\|x_{i}-x_{j}{\|}^{2}}{Te}}.$$

Here, we define the *L* as the equation $L=D^{k}-L^{k}$. $D^{k}$ is a diagonal matrix whose entries are column (or row, since Sis symmetric) sums of $L^{k}$. For convenience, the neighbor number used in our graph is denoted as *r*.

### 2.3. Variational Expectation Maximization Inference

Our scheme for estimating the data cluster depends mainly on our capability to infer the posterior distribution. We solve this using variational expectation maximization inference.

Unlike the conventional expectation maximization algorithm, the posterior probability in our model will be estimated via the variational inference, and then, we optimize the MoG parameter by maximizing the lower bound with the fixed variational parameter. Following the general variational inference framework, we firstly give the Evidence Lower BOund (ELBO) for the SimDPM with the truncated stick-breaking process (when applying this process, the maximum cluster number *∞* is truncated to *K*) [37].

(4) $$\begin{array}{cc} \log(p\left(X|\alpha ,\lambda ,\tilde{\Theta }\right)-\lambda_{R}R\geq & E_{q}\left[\log p\left(\Theta^{*}|\lambda \right)\right]+\sum_{n=1}^{N}E_{q}\left[\log p\left(z_{n}^{\left(1\right)},z_{n}^{\left(2\right)}|V\right)\right]+\sum_{n=1}^{N}E_{q}\left[\log p\left(x_{n}|\Theta^{*},z_{n}^{\left(1\right)}\right)\right]\\ & -E_{q}\left[\log q\left(v,\Theta^{*},z^{\left(1\right)},z^{\left(2\right)}\right)\right]+\sum_{n=1}^{N}E_{q}\left[\log p\left(x_{n}|\tilde{\Theta },z_{n}^{\left(2\right)}\right)\right]\\ & +E_{q}\left[\log p\left(v|\alpha \right)\right]-\lambda_{R}R, \end{array}$$

where $X={\left\{x_{n}\right\}}_{n=1}^{N}$ is the observation sample, $p(\Theta^{*}|\lambda )$ is a normal-Wishart distribution with hyper parameters $\lambda =(u_{0},c_{0},W_{0},v_{0})$ and $p(x_{n}|\tilde{\Theta })$ is the constrained MoG distribution parameterized by $\tilde{\Theta }={\left\{{\tilde{\theta }}_{k}\right\}}_{k=1}^{K}$ where ${\tilde{\theta }}_{k}=\left\{{\tilde{u}}_{k},{\tilde{\delta }}_{k},{\tilde{\pi }}_{k}\right\}$, $p(x_{n}|\Theta^{*})$ is a single Gaussian distribution. $z^{\left(1\right)}={\left\{z_{n}^{\left(1\right)}\right\}}_{n=1}^{N}$ and $z^{\left(2\right)}={\left\{z_{n}^{\left(2\right)}\right\}}_{n=1}^{N}$ are the indicator variables sampled from the categorical distribution $p(z_{n}^{\left(1\right)},z_{n}^{\left(2\right)}|v)$. Following the factorized family variational inference [37], which can make the posterior distribution computable, *q* can be expressed as:

(5) $$\begin{array}{cc} q(v,\Theta^{*},z^{\left(1\right)}, & z^{\left(2\right)})=\prod_{k=1}^{K-1}q_{\gamma_{k}}\left(v_{k}\right)\prod_{k=1}^{K}q_{\tau_{k}}\left(\theta_{k}^{*}\right)\prod_{n=1}^{N}q_{{s\phi }_{n}}\left(z_{n}^{\left(1\right)}\right)\prod_{n=1}^{N}q_{{(1-s)\phi }_{n}}\left(z_{n}^{\left(2\right)}\right), \end{array}$$

where $q_{\gamma_{k}}\left(v_{k}\right)$ is the Beta distribution with $\gamma_{k}=\left\{\gamma_{k,1},\gamma_{k,2}\right\}$ and $q_{\tau_{t}}\left(\theta_{t}^{*}\right)$ is a normal-Wishart distribution with the parameter $\tau_{k}=\left\{u_{k},c_{k},W_{k},v_{k}\right\}$. For $q_{s\phi_{n}}\left(z_{n}^{\left(1\right)}\right)$ and $q_{\left(1-s\right)\phi_{n}}\left(z_{n}^{\left(2\right)}\right)$, we denote it as two categorical distributions with parameter $\phi_{n}={\left\{\phi_{n,k}\right\}}_{k=1}^{K}$ ($\Phi ={\left\{\phi_{n}\right\}}_{n=1}^{N}\in R^{N\times K}$). *s* is the tradeoff parameter.

For derivation convenience, we denote ELBO as $L(\gamma ,\tau ,\Phi ,\tilde{\Theta })$. By using this inequality relaxation, we note that learning the model and estimating the model parameters are altered to maximize the following equation.

$$\begin{array}{c} \underset{\left\{\gamma_{k},u_{k},c_{k},W_{k},v_{k},{\tilde{u}}_{k},{\tilde{\delta }}_{k},{\tilde{\pi }}_{k}\right\}}{\arg\max}L\left(\gamma ,\tau ,\Phi ,\tilde{\Theta }\right)-\lambda_{R}R\\ s.t.\frac{1}{2}{\left({\tilde{u}}_{k,m}-{\tilde{u}}_{k,m-1}\right)}^{T}{\tilde{\delta }}_{k,m}^{-1}\left({\tilde{u}}_{k,m}-{\tilde{u}}_{k,m-1}\right)\epsilon ,\;m1,\\ R=\sum_{k=1}^{\infty }p{\left(k|X\right)}^{T}Lp\left(k|X\right). \end{array}$$

We also notice that, since we have truncated the maximum cluster number to *K*, the penalty term *R* is altered to be $R=\sum_{k=1}^{K}p{\left(k|X\right)}^{T}Lp\left(k|X\right)$.

Variational E-step: In the variational inference framework, the variational parameter can be estimated by maximizing the lower bound of likelihood function logp(X|α,λ) with the coordinate ascent algorithm.

For $\phi_{n,k}$ in ${\left\{\phi_{n,k}\right\}}_{k=1}^{K}$, note that this is a constrained maximization since $\sum_{k=1}^{K}\phi_{n,k}=1$, and the probability p(k|X) can be approximated by the variational parameter $\Phi_{.,k}$. To solve this problem, we use an auxiliary variable $A_{k}=\Phi_{.,k}$ where $A_{k}\in R^{N}$ and form the Lagrangian by isolating the terms in ELBO, which contain $\phi_{n,k}$ as:

(6) $$\begin{array}{cc} \underset{\phi_{n}}{\arg\max} & L\left(\gamma ,\tau ,\Phi ,\tilde{\Theta }\right)+\lambda_{L}\left(\sum_{k=1}^{K}\phi_{n,k}-1\right)-\lambda_{R}\sum_{k=1}^{K}A_{k}^{T}LA_{k},\\ & s.t.\;A_{k}=\Phi_{.,k}, \end{array}$$

where $\lambda_{L}$ is a Lagrangian multiplier and $\lambda_{R}$ is a penalty parameter.

Fix $A_{k}$ to update $\Phi_{.,k}$. The updating rule for $\phi_{n,k}$ can be achieved by taking the derivation.

(7) $$\begin{array}{cc} \log\phi_{n,k}\propto & sE_{q}\left[\log p\left(x_{n}|\theta_{k}^{*}\right)\right]+\left(1-s\right)\log p\left(x_{n}|{\tilde{\theta }}_{k}\right)\\ & +\sum_{jk,n}^{}\left(\Psi \left(\gamma_{j,2}\right)-\Psi \left(\gamma_{j,1}+\gamma_{j,2}\right)\right)\\ & +\Psi \left(\gamma_{k,1}\right)-\Psi \left(\gamma_{k,1}+\gamma_{k,2}\right). \end{array}$$

We now fix Φ to update $H_{k}$.

(8) $$\begin{array}{c} \underset{A_{k}}{\arg\min}A_{k}^{T}LA_{k}+\lambda_{A}\|A_{k}-\Phi_{.,k}^{old}{\|}_{2}^{2}\\ ⟹A_{k}=\Phi_{.,k}=\lambda_{H}{\left(\lambda_{A}I+L\right)}^{-1}\Phi_{.,k}^{old}, \end{array}$$

where $\lambda_{A}$ is the penalty parameter. For the other variational parameter, we can attain the following closed-form solutions when taking the derivation of the previous proposed ELBO function and setting it to zero:

(9) $$\begin{array}{c} \gamma_{k,1}=1+\sum_{i=1}^{N}\phi_{i,k},\\ \gamma_{k,2}=\alpha +\sum_{i=1}^{N}\sum_{jk}\phi_{i,j}, \end{array}$$

(10) $$\begin{array}{c} c_{k}=c_{0}+N_{k},\\ v_{k}=v_{0}+N_{k}, \end{array}$$

(11) $$\begin{array}{c} u_{k}=\frac{1}{c_{k}}\left(c_{0}u_{0}+N_{k}{\bar{x}}_{k}\right),\\ W_{k}^{-1}=W_{0}^{-1}+N_{k}S_{k}+\frac{c_{0}N_{k}}{c_{0}+N_{k}}\left({\bar{x}}_{k}-u_{0}\right){\left({\bar{x}}_{k}-u_{0}\right)}^{T} \end{array}$$

where $N_{k}$, S${}_{k}$ and $\overset{-}{x}$${}_{k}$ can be estimated as follows:

(12) $$\begin{array}{c} N_{k}=\sum_{n=1}^{N}s\phi_{n,k},\\ {\bar{x}}_{k}=\frac{s}{N_{k}}\sum_{n=1}^{N}\phi_{n,k}x_{n},\\ S_{k}=\frac{s}{N_{k}}\sum_{n=1}^{N}\phi_{n,k}\left(x_{n}-{\bar{x}}_{k}\right){\left(x_{n}-{\bar{x}}_{k}\right)}^{T}. \end{array}$$

For the prior parameters $u_{0},c_{0},W_{0},v_{0}$, we use them in a non-informative manner to make them influence as little as possible the inference of the variational posterior distributions. For the other variational parameters, we initialize them in a random way.

Variational M-step: To optimize the lower bound parameter $\tilde{\theta }$, we apply the EM framework again, in which we introduce an auxiliary posterior variable $q(k,m|x_{n})$ and Jensen’s inequality [37].

(13) $$\begin{array}{c} L\left(\gamma ,\tau ,\Phi ,\tilde{\Theta }\right)+H\sum_{m=2}^{M}\{{\left({\tilde{u}}_{k,m}-{\tilde{u}}_{k,m-1}\right)}^{T}{\tilde{\delta }}_{k,m}^{-1}\left({\tilde{u}}_{k,m}-{\tilde{u}}_{k,m-1}\right)\}\\ \geq C+\sum_{n=1}^{N}\sum_{k,m=1}^{K,M}\{\left(1-s\right)\phi_{n,k}q\left(k,m|x_{n}\right)\log\frac{{\tilde{\pi }}_{k,m}N\left(x_{n}|{\tilde{u}}_{k,m},{\tilde{\delta }}_{k,m}\right)}{q(k,m|x_{n})}\}\\ +H\sum_{m=2}^{M}\{{\left({\tilde{u}}_{k,m}-{\tilde{u}}_{k,m-1}\right)}^{T}{\tilde{\delta }}_{k,m}^{-1}\left({\tilde{u}}_{k,m}-{\tilde{u}}_{k,m-1}\right)\}, \end{array}$$

where *C* is a constant value with no respect to $\tilde{\Theta }$ in $L(\gamma ,\tau ,\Phi ,\tilde{\Theta })$. By using the inequality relaxation, the variational M-step can be reformulated as the optimization problem:

(14) $$\begin{array}{cc} \max & \{\sum_{n=1}^{N}\phi_{n,k}q\left(k,m|x_{n}\right)\log\frac{{\tilde{\pi }}_{k,m}N\left(x_{n}|{\tilde{u}}_{k,m},{\tilde{\delta }}_{k,m}\right)}{q(k,m|x_{n})}\\ & +H{\left({\tilde{u}}_{k,m}-{\tilde{u}}_{k,m-1}\right)}^{T}{\tilde{\delta }}_{k,m}^{-1}\left({\tilde{u}}_{k,m}-{\tilde{u}}_{k,m-1}\right)\\ & +\lambda_{L}\left(\sum_{m=1}^{M}q\left(k,m|x_{n}\right)-1\right)\}, \end{array}$$

where $\lambda_{L}$ and *H* are the Lagrangian multipliers. We therefore achieve the following closed-form solution by taking the derivative and setting the lower bound of (14) to zero:

(15) $$\begin{array}{c} {\tilde{u}}_{k,1}=\frac{\sum_{n=1}^{N}\phi_{n,k}q\left(k,1|x_{n}\right)x_{n}}{\sum_{n=1}^{N}\phi_{n,k}q\left(k,1|x_{n}\right)}; \end{array}$$

when *m* is greater than one, we have:

(16) $$\begin{array}{c} {\tilde{u}}_{k,m}=\frac{\sum_{n=1}^{N}\phi_{n,k}q\left(k,m|x_{n}\right){\tilde{\delta }}_{k,m-1}x_{n}-H{\tilde{u}}_{k,m-1}}{\sum_{n=1}^{N}\phi_{n,k}q\left(k,m|x_{n}\right)-H}. \end{array}$$

Similar to the mean parameter ${\tilde{u}}_{k,m}$, for ${\tilde{\delta }}_{k,m}$, we have:

(17) $$\begin{array}{c} {\tilde{\delta }}_{k,m}=\frac{T_{k,m}}{\sum_{n-1}^{N}\phi_{n,k}q\left(k,m|x_{n}\right)},m1, \end{array}$$

where:

$$\begin{array}{cc} T_{k,m} & =-H\left({\tilde{u}}_{k,m}-{\tilde{u}}_{k,m-1}\right){\left({\tilde{u}}_{k,m}-{\tilde{u}}_{k,m-1}\right)}^{T}\\ & +\sum_{n=1}^{N}\phi_{n,k}q\left(k,m|x_{n}\right)\left(x_{n}-{\tilde{u}}_{k,m}\right){\left(x_{n}-{\tilde{u}}_{k,m}\right)}^{T}, \end{array}$$

since the constraint does not exist in the components where m2, the updating rule for m=1 is a little different.

(18) $$\begin{array}{c} {\tilde{\delta }}_{k,m}=\frac{\sum_{n=1}^{N}\phi_{n,k}q\left(k,m|x_{n}\right)\left(x_{n}-{\tilde{u}}_{k,m}\right){\left(x_{n}-{\tilde{u}}_{k,m}\right)}^{T}}{\sum_{n=1}^{N}\phi_{n,k}q\left(k,m|x_{n}\right)}. \end{array}$$

For the computation of $\pi_{k,m}$, we have:

(19) $$\begin{array}{c} {\tilde{\pi }}_{k,m}=\frac{\sum_{n=1}^{N}\phi_{n,k}q\left(k,m|x_{n}\right)}{\sum_{m}^{M}\sum_{n=1}^{N}\phi_{n,k}q\left(k,m|x_{n}\right)}. \end{array}$$

The computation of $q(k,m|x_{n})$ will be identical to the standard mixture of Gaussians model learning algorithm [37].

### 2.4. Agorithm

**Figure 3:**
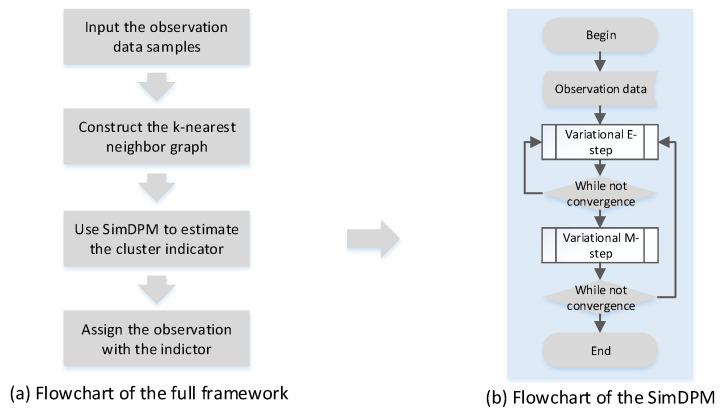
Illustration of the framework flowchart. (**a**) shows the flowchart of the framework; (**b**) demonstrates the flowchart of SimDPM.

The full learning and inference algorithm is summarized in Algorithm 1. The flowchart of our proposed framework is demonstrated in Figure 3. Below, we analyze the computational complexity.
**Algorithm 1** Semi-supervised DPM clustering algorithm.**Require:**   unlabeled dataset $X_{u}$.**Ensure:**   variational parameters, ${\left\{\gamma_{k},\tau_{k},\phi_{k}\right\}}_{k=1}^{K}$, and model parameter, ${\left\{\tilde{\theta_{k}}\right\}}_{k=1}^{K}.$1:Construct the *k*-nearest neighbor graph $L^{k}$ and *L*. Initialize the variational parameter randomly.2:**while** not convergent **do**3: **Expectation step:**4: **while** not convergent **do**5:  **for** all n,k **do**6:   Update the variational parameters $\left\{\;\phi_{n,k}^{old}\right\}$ using (7).7:   Update the variational parameters $\left\{\nu_{k},\;\phi_{k},\;c_{k},\;u_{k},\;W_{k}^{-1},\;v_{k}\right\}$ using (9), (10) and (11).8:  **end for**9:  Update the variational parameters $\left\{\;\phi_{n,k}\right\}$ using (8).10: **end while**11: **Maximization step:**12: **for** k=1;k≤K;k=k+1 **do**13:  Update ${\tilde{u}}_{k,1}$ and ${\tilde{\delta }}_{k,1}$ with (15) and (18).14:  **for** m=2;m≤M;m=m+1 **do**15:   Update ${\tilde{u}}_{k,m}$, ${\tilde{\delta }}_{k,m}$ and ${\tilde{\pi }}_{k,m}$ using (16), (17) and (19).16:  **end for**17: **end for**18: $q\left(k,m|x_{n}\right)\leftarrow \frac{{\tilde{\pi }}_{k,m}N\left(x_{n}|{\tilde{u}}_{k,m},{\tilde{\delta }}_{k,m}\right)}{\sum_{m=1}^{M}{\tilde{\pi }}_{k,m}N\left(x_{n}|{\tilde{u}}_{k,m},{\tilde{\delta }}_{k,m}\right)}$19:**end while**

**Algorithm complexity**: Suppose that we have *N* samples, each sample has *D* dimensions. The maximum cluster number in our experiment is *K*. Expectation step converges after running *T_e_* times. The whole algorithm converges after *T* times. From the derivation, we know that the main computation lies on the Equations (7), (8) and (11), in which we need to calculate the inverse and determinant of the matrix. For Equations (7) and (11), we need *O*(*K* · *D*^3^). For Equation (8), we need *O*(*N*^3^). Another major computation is the Equations (16) and (17), which takes the computational complexity of *O*((*M* − 1) · *K* · *N* · *D*^2^). According to the debates, we know that the whole algorithm computational complexity is *O*(*T* · (*T_e_* · (*N*^3^ + *K* · *D*^3^) + (*M* − 1) · *K* · *N* · *D*^2^)).

For the space complexity, the main cost is the variational parameters which takes *O*(*K* · *D*^2^ + *N* · *K*). Another cost is the MoG parameters which needs *O*(*M* · *K* · *D*^2^). Then, the total space complexity is *O*(*K* · *D*^2^ + *N* · *K* + *M* · *K* · *D*^2^).

## 3. Results

To demonstrate the usefulness of the proposed manifold model, we tested our method on both synthetic and real-world datasets and compared it with the following methods:

1. Original Dirichlet Process Mixture (DPM) model [38].
2. Affinity Propagation (AP) clustering [39].
3. A Dirichlet process-based linear manifold clustering method, DP-space [28].
4. Density-based Clustering algorithm by Fast Search and Find of Density Peaks (CFSFDP) [40].
5. Another category is the clustering method, which needs to specify the class number, *K*-means, LRR [17] and LatLRR [41].

Clustering accuracy in our experiment was measured through Normalized Mutual Information (NMI) [32]. Suppose $U=\{U_{1},U_{2},U_{3},\ldots ,U_{\left|U\right|}\}$ denotes the real cluster labels obtained from the ground truth and $V=\{V_{1},V_{2},V_{3},\ldots ,V_{\left|V\right|}\}$ obtained from a clustering algorithm. |U| and |V| denote the cluster number. Then, a mutual information metric between *U* and *V* can be defined as:

(20) $$\begin{array}{c} MI\left(U,V\right)=\sum_{i=1}^{\left|U\right|}\sum_{j=1}^{\left|V\right|}P\left(i,j\right)log\left(\frac{P(i,j)}{P(i)P(j)}\right) \end{array}$$

where P(i) and P(j) are the probability that a sample picked at random falls into class $U_{i}$ or $V_{j}$ and P(i,j) denotes the probability that a sample falls into both classes $U_{i}$ and $V_{j}$. The Normalized Mutual Information (NMI) then can be defined as:

(21) $$\begin{array}{c} NMI\left(U,V\right)=\frac{MI(U,V)}{\sqrt{\left(H(U)H(V)\right)}} \end{array}$$

where H(U) and H(V) denote the entropy.

Experimental setup: In our experiment, we ran every algorithm 10 times and report the average accuracy. The parameters of the SimDPM algorithm were selected using the ground-truth labels of less than 40% according to the clustering accuracy. The default value for α and the maximum cluster number *K* were set at 20 and 30. The other variational parameters were initialized randomly except $u_{k}$ and $W_{k}$, for which we used the mean and covariance of the observation data to initialize. All our algorithms were implemented in MATLAB R2016a on a DELL Precision Workstation with 8.00 G RAM and a Xeon(R) E3 CPU.

For the original DPM, we used the α=20 and set the other variational parameters randomly. When operating the DP-space, we used λ and *s* from the values, as this was suggested in the original codes, and we selected this by using 30% ground-truth labels. The parameters used in LatLRR and LRR were that α=1, β=1.4 and λ=4. For CFSFDP, we chose the determination points that were significantly different from the other points in the decision graph. In the setting of AP, we used the preference value as a scalar one. Both CFSFDP and AP used the *K*-nearest neighbor graph as the similarity matrix.

### 3.1. Synthetic Dataset

**Figure 4:**
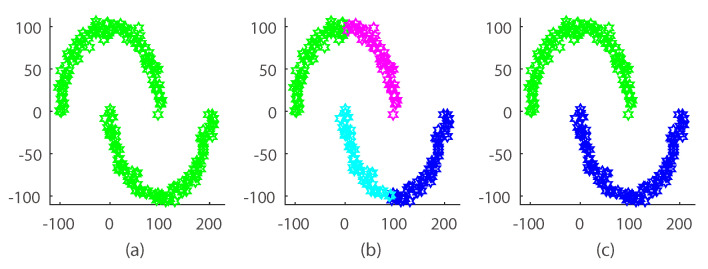
Illustration of SimDPM and the original DPM clustering result on a synthetic dataset. (**a**) demonstrates the original dataset with no label; (**b**,**c**) are the original DPM clustering result and SimDPM clustering result. Different color means different cluster.

In this section, we evaluate our SimDPM model on a synthetic dataset. We show the results in Figure 4. Clearly, there are two patterns.

Visual comparison on synthetic hybrid data shows that SimDPM performed better than the traditional DPM model. In our result, Figure 4b shows the result using the original variational DPM. As can be seen, the original DPM tended to partition the synthetic dataset in a hard manner. Our manifold method yielded an ideal clustering result. The reason is that our model handled the dataset with a Gaussian expanding distribution and reserved the local geometrical structure of the data space by applying a *k*-nearest neighbor graph.

### 3.2. Real Dataset

**Figure 5:**
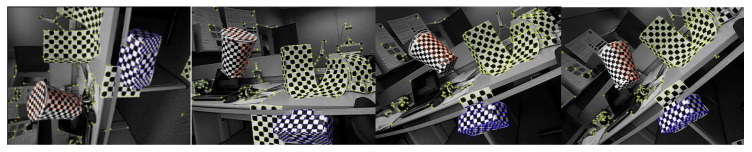
Illustration of the Hopkin155 dataset.

**Figure 6:**
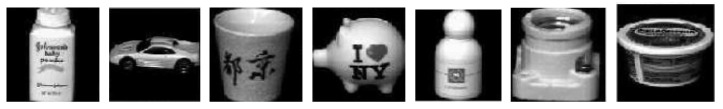
Illustration of the coil20 dataset.

**Figure 7:**
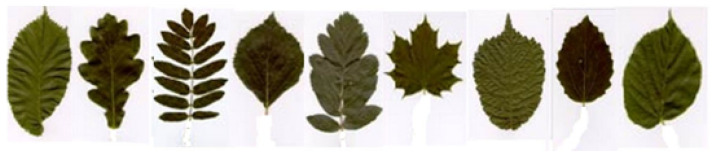
Leaf samples from the leaf dataset.

(a) Motion segmentation: Motion segmentation usually refers to the task of separating the movements of multiple rigid-body objects from video sequences. Linear manifold clustering methods are popular in this task [42]. In our experiment, we used the Hopkin155 dataset [43] and cast it into a general multi-manifold clustering task. We show some samples in Figure 5. According to the dataset itself, we divided the universal set into checkerboard and others [43], in which each contained 26 and nine subsets. For the checkerboard dataset, we separated it into the Linear manifold dataset (L) and Non-Linear manifold dataset (Non-L) according to the 3D projection of PCA. When applying our algorithm, we projected point trajectories into 10D features. The clustering result and the estimated cluster number are presented in Table 2 and Table 3. As can be seen, our proposed method performed the best on the Non-L dataset. On the others and L dataset, DP-space was the first best, and our method was the second best. For the estimated cluster number, we can observe that our model could produce the suitable cluster size compared with the ground truth.

(b) Coil20 image dataset: The coil20 [44] image database is a popular manifold database containing 20 objects from the Columbia university image library. Some image samples are demonstrated in Figure 6. Each image is taken from five degrees apart as the object is rotated on a turntable. Thus, each object in coil20 has 72 images. The size of the object is 128 × 128, with 256 grey levels per pixel. In our experiment, each image was firstly represented by a 128 × 128 dimensional vector, and then, we projected it into a 10D feature using the PCA method. To test the general clustering performance, we used five coil20 subsets. For the overall testing, we also gave the universal dataset (Dataset 20). The clustering result and the estimated cluster number are demonstrated in Table 4 and Table 5. From the result, we know that our method consistently outperformed the DP-based algorithms such as DP-space and DPM. When comparing with the other methods, our method was the first or the second best, especially compared with the approaches that do not need to specify the cluster number.

(c) Swedish leaf image dataset: The Swedish dataset introduced in [45] consists of 1125 leaves of 15 species with 75 images per species. In this dataset, we firstly extracted the outer contour and then achieved the contour features by applying the Fourier transform [46]. Every leaf in our experiments was represented as a 10-dimensional feature. Some samples are shown in Figure 7.

Similar to the coil20 dataset, we demonstrated the efficiency on five subsets and the universal dataset. We ran every algorithm in our experiment 10 times, and took the accuracy by averaging the 10 results. The experimental results are demonstrated in Table 6 and Table 7, which present some observations: (1) compared with the original DPM, the improvement of the clustering accuracy (average 0.07) was lower than the improvement in the coil20 dataset (average 0.05); (2) the cluster number was consistently increasing as the ground truth cluster number was increasing.

From the experimental results in Table 2, Table 3, Table 4, Table 5, Table 6 and Table 7, we can draw some points as follows.

- The proposed method obtained the highest clustering accuracy especially on the Non-L and coil20 dataset compared with the non-prespecified cluster number methods, which validates the effectiveness of our non-linear assumption.
- DP-space performed better than our method on the L and others dataset, the reason being that DP-space has a prior structure assumption, which introduces additional manifold geometric information.
- LRR, LatLRR and *K*-means outperformed our algorithm on some coil20 and leaf subdatasets, the reason being that our method needed to estimate the cluster number along with clustering. This made our algorithm hard to optimize.
- Compared to the coil20 and leaf dataset, our method achieved an all-around performance boosting on the motion segmentation dataset; this is because the simple clustering task (the linear manifold has only three classes) was easy for our algorithm to optimize and model.
- Compared to the leaf dataset, our method achieved a better clustering performance boosting on the coil20 dataset. The reason is that coil20 is a well-defined manifold dataset, in which the structure among samples is easy to capture by the graph Laplacian.
- Our manifold model consistently produced the suitable cluster number with the increasing of the data cluster size, which indicates that our model could provide a flexible model size when fitting different datasets.

### 3.3. The Effect of the Algorithm Parameters

**Figure 8:**
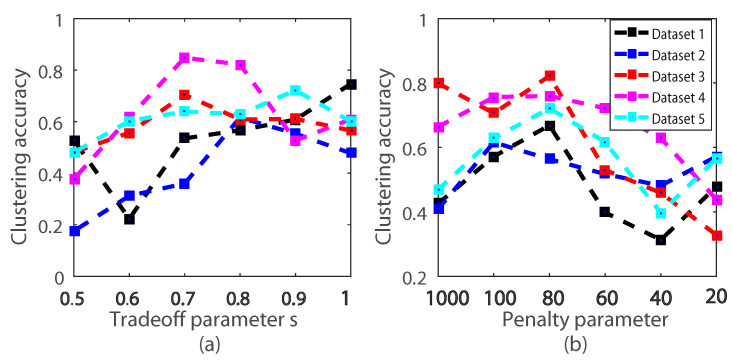
Illustration of the clustering accuracy with different *s* and $\lambda_{A}$ on five hopkin155 datasets. (**a**) is the clustering accuracy with different *s*. (**b**) is the clustering accuracy with different $\lambda_{A}$.

In this section, we firstly investigate the effects of the parameters $\lambda_{A}$ and *s* on the Non-L dataset. More specifically, in the experiment, when one parameter is being tuned, the value of the other parameter is fixed. The parameters $\lambda_{A}$ and *s* were sampled from {1000,100,80,60,40,20} and {1,0.9,0.8,0.7,0.6,0.5}. We show the clustering accuracy in Figure 8.

**Figure 9:**
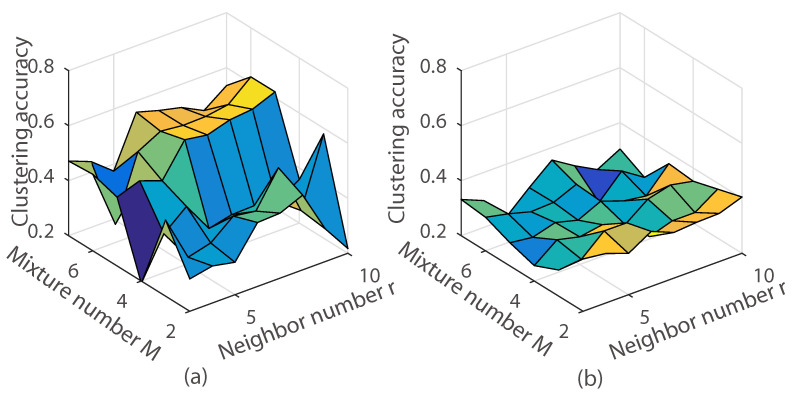
Illustration of the clustering accuracy with different *M* and the neighbor number *r* using the *k*-nearest neighbor graph on the leaf dataset. (**a**) Subdataset of leaf with 6 classes; (**b**) The leaf full dataset.

**Figure 10:**
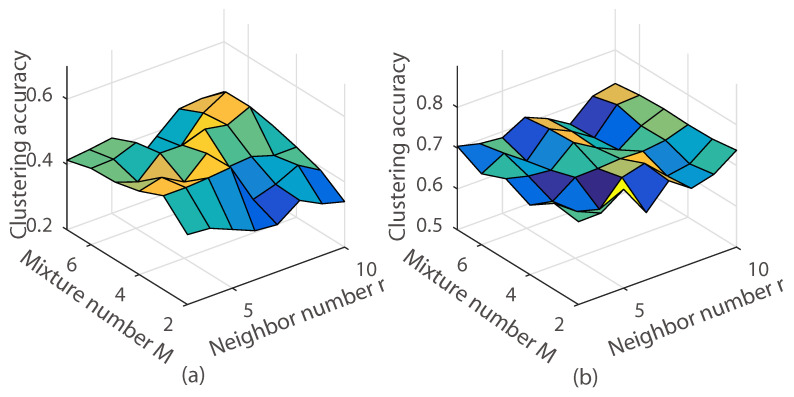
Illustration of the clustering accuracy with different *M* and the neighbor number *r* using the *k*-nearest neighbor graph on the coil20 dataset. (**a**) Subdataset of coil20 with 6 classes; (**b**) The coil20 full dataset.

As we can see in Figure 8, experimental results indicate that the proposed model was sensitive to $\lambda_{A}$. Empirically, the best clustering accuracy was achieved when $\lambda_{A}=100$. We also observed that our method achieved the best clustering accuracy when s=0.7,0.8. This reveals that the MoG had improved the clustering accuracy. Besides, we measured the clustering accuracy with the different *M* and the neighbor number *r* using the *k*-nearest neighbor graph. The clustering accuracy is demonstrated in Figure 9 and Figure 10. As can be seen, the clustering accuracy achieved the best performance on the leaf subdataset and the coil20 dataset when the neighbor number r={5,6,7,8,9,10}. Clustering accuracy increased along with the increasing of the *M* in the subdataset of the coil20 dataset and leaf dataset. Unlike the subdataset, parameter *M* and the neighbor number *r* had little effect on the full dataset of coil20 and leaf. The reason is that our model is a non-convex model, and the complicated dataset led to a much more complicated optimization.

## 4. Discussion

Compared to the previous linear manifold and geodesic mixture models, our theoretical analysis has shown that our method is a prespecified manifold and cluster number-free model. This is because we use a DP prior to generate the cluster indicator with the suitable cluster number and use the MoG and *K*-nearest neighbor graph to capture the submanifold rather than using a predefined manifold. Additionally, compared with different multi-manifold clustering methods with prespecified manifolds and cluster numbers like DP-space, LRR and LatLRR, our method has shown superior performance on the general manifold clustering task (coil20 and leaf dataset). This indicates our method can fill the research gap we have mentioned in the Introduction.

Although our method can handle the problems we have mentioned (estimating the cluster number and handling the general manifold clustering task), limitations still exist. That is, our method is not a full Bayesian framework. Thereby, some parameters should be tuned manually. This may be unacceptable in some real applications. Moreover, we note that MoG and the Gaussian distribution are sensitive to the dimension of the data. In future work, we will explore a full generative model, in which the parameters can be generated by using some Bayesian priors. Since our approach is sensitive to the dimension, we will also explore certain methods to integrate the dimension reduction method and the manifold clustering.

## 5. Conclusions

In this paper, we have proposed a nonparametric generative model to handle the manifold dataset with no prespecified cluster number and manifold distribution. In the course of the theoretical and experimental analysis, we have demonstrated that MoG can extend the application scope of the original DPM and can significantly improve the clustering accuracy compared to the previous proposed method. However, to be frank, the proposed method can only partially handle the problem we state in the Introduction due to the facts that: (1) MoG, the mean constraint and *K*-nearest neighbor graph are hard to optimize when we incorporate them into the DP framework; this can be observed when we use it in the coil20 and full leaf dataset; (2) the DP prior has a limitation when generating the suitable cluster number.

## Figures and Tables

**Table 1:** The main notations and descriptions.

Notations	Descriptions
λ	Hyper parameter $u_{0},c_{0},W_{0},\nu_{0}$ of normal-Wishart
*M*	The mixture number
*N*	Number of the observation samples
$\theta_{k}^{*}$	Gaussian parameter u,δ
${\tilde{\theta }}_{k}$	MoG parameter ${\tilde{u}}_{k},{\tilde{\delta }}_{k},{\tilde{\pi }}_{k}$
*s*	Tradeoff parameter
*K*	The maximum cluster number
α	Parameter of the Beta distribution
$z_{n}^{\left(1\right)}$	Class indicator of a Gaussian distribution
$z_{n}^{\left(2\right)}$	Class indicator of the MoG distribution
*X*	Unlabeled dataset
$\gamma_{k}$	Variational parameter $\gamma_{k,1},\gamma_{k,2}$
$\tau_{k}$	Variational parameter of normal-Wishart
$\Phi_{n}$	Variational parameter of categorical distribution
*A*	An auxiliary parameter that equals Φ
$\lambda_{A}$	Penalty parameter used in the graph Laplacian
*L*	Graph Laplacian
$L^{k}$	*k*-nearest neighbor graph
$D^{k}$	Diagonal matrix whose entries are column sums of $L^{k}$
*R*	Posterior penalty term with graph Laplacian
*r*	Neighbor number used in the graph Laplacian

**Table 2:** Clustering accuracy on the Hopkin155 dataset with 3 motions. AP, Affinity Propagation; CFSFDP, Density-based Clustering algorithm by Fast Search and Find of Density Peaks; LRR, Low-Rank Representation. Bolded numbers denote the highest clustering accuracy.

Method	Checkerboard			Others
	L	Non-L	Average	
SimDPM	0.80	**0.73**	**0.79**	0.83
DPM [38]	0.42	0.37	0.41	0.45
DP-space [28]	**0.84**	0.48	0.78	**0.94**
AP [39]	0.29	0.32	0.29	0.31
CFSFDP [40]	0.40	0.19	0.36	0.47
K-means	0.48	0.48	0.49	0.47
LRR [17]	0.51	0.33	0.48	0.33
LatLRR [41]	0.52	0.31	0.47	0.34

**Table 3:** The estimated cluster number on the Hopkin155 dataset with 3 motions. L, Linear.

Method	Checkerboard			Others
	L	Non-L	Average	
Ground truth	3.00	3.00	3.00	3.00
The estimated cluster number	3.33	3.60	3.10	3.09

**Table 4:** Clustering accuracy on the coil20 dataset. Bolded numbers denote the highest clustering accuracy.

Method	Subdataset					20
	2	4	6	8	10	
SimDPM	**0.30**	0.55	0.56	**0.60**	**0.69**	0.72
DPM [38]	0.29	0.42	0.50	0.53	0.57	0.69
DP-space citewang2015dp	0.01	0.34	0.10	0.19	0.10	0.26
AP [39]	0.12	0.22	0.18	0.11	0.25	0.36
CFSFDP [40]	0	0.57	0.57	0.53	0.46	0.42
K-means	0	0.52	0.46	0.57	0.59	**0.73**
LRR [17]	0.11	**0.62**	0.56	0.47	0.52	0.70
LatLRR [41]	0	0.57	**0.57**	0.48	0.50	0.58

**Table 5:** The estimated cluster number on the coil20 dataset.

Method	Subdataset					20
	2	4	6	8	10	
Ground truth	2.0	4.0	6.0	8.0	10.0	20.0
The estimated cluster number	3.1	5.0	6.3	6.7	11.3	21.7

**Table 6:** Clustering accuracy on the leaf dataset. Bolded numbers denote the highest clustering accuracy.

Method	Subdataset					15
	2	4	6	8	10	
SimDPM	0.29	**0.62**	0.46	**0.50**	**0.54**	**0.38**
DPM [38]	0.26	0.43	0.45	0.49	0.51	0.34
DP-space [28]	0.49	0.33	0	0	0	0.03
AP [39]	0	0.03	0.11	0	0.02	0
CFSFDP [40]	0.17	0.56	0.24	0.28	0.42	0
K-means	0.45	0.39	**0.61**	0.50	0.44	0.22
LRR [17]	**0.76**	0.45	0.48	0.33	0.40	0.22
LatLRR [41]	0.65	0.44	0.32	0.20	0.41	0.23

**Table 7:** The estimated cluster number on the leaf dataset.

Method	Subdataset					15
	2	4	6	8	10	
Ground truth	2.0	4.0	6.0	8.0	10.0	15.0
The estimated class number	4.7	6.3	10.2	14.5	16.2	20.2
